# *Big and Mini*: A Promising Intergenerational Program for Social Connections

**DOI:** 10.3390/ijerph19084566

**Published:** 2022-04-11

**Authors:** Ling Xu, Noelle L. Fields, Zhirui Chen, Allen Zhou, Aditi Merchant, Anthony Zhou

**Affiliations:** 1 School of Social Work, University of Texas at Arlington, 211 S. Cooper Street, Arlington, TX 76019, USA; noellefields@uta.edu (N.L.F.); zhirui.chen@mavs.uta.edu (Z.C.); 2 Department of Electrical & Computer Engineering, University of Texas at Austin, Austin, TX 78712, USA; allen.zhou101@utexas.edu; 3 Department of Biomedical Engineering, University of Texas at Austin, Austin, TX 78712, USA; aditi.merchant@utexas.edu; 4 Department of Computer Science, Columbia University, New York, NY 10027, USA; az2681@columbia.edu

**Keywords:** intergenerational program, *Big and Mini*, COVID-19, loneliness, social connections

## Abstract

Introduction: To help older adults cope with loneliness during COVID-19, a weekly, telephone-based intergenerational program called “*Big and Mini*” was created in April 2020 to link young and older adults together. As part of an evaluation of *Big and Mini*, a survey with both close and open-ended questions was sent to participants. Methods: A total of 63 Bigs and 53 Minis completed the survey. Their stress compared to before COVID-19, loneliness, life satisfaction, intergenerational closeness, and satisfaction with the program were measured for participants. Descriptive, bivariate correlation and conventional content analyses were conducted. Results: On average, Bigs and Minis had participated in the program for 3.73 and 3.49 months, respectively. Approximately half of the Bigs (47.6%) and Minis (52.8%) felt the same stress level compared to before COVID-19. A few participants felt “less stressed” compared to before COVID -9 (14.3 and 7.5%, respectively, for Bigs and Minis). All participants reported medium levels of loneliness, high levels of satisfaction with life, satisfaction with the program, and intergenerational closeness. Content analysis suggested that the reasons to join or expectations of the program were friendship, mutually beneficial intergenerational connections, and coping with loneliness. Conclusions: The *Big and Mini* program offers a promising approach with mutual benefits for participants. Strategies to improve the program and implications for intergenerational programs are presented.

## 1. Introduction

Physical or mental health impairment(s) in later life may increase an individual’s risk of isolation and limit their support networks. According to the National Council on Aging, more than 6.7 million adults are aged 65 and older, and about 17 percent of this older generation live in social or geographical isolation, or both [1]. During COVID-19, prolonged periods of social distancing may result in negative effects for older adults long after quarantining ends [2]. Overall, older adults are at higher risk for contracting COVID-19 and reported feeling isolated, lonely, and stressed as the pandemic progressed [3].

Social isolation and loneliness are a serious social and public health problem affecting significant numbers of older adults aged 65 and older and their health [4]. Individuals in later life may experience life situations that place them at higher risk for social isolation and loneliness. However, social isolation and loneliness are not an inevitable, normal part of aging. A growing body of research suggests that meaningful social connections can reduce social isolation and loneliness [5]. Due to social and physical distancing requirements and recommendations for sheltering-in-place during COVID-19, it is strongly argued that increased efforts should be made to keep social connections among older adults to help them cope with loneliness [6].

Increased risk of loneliness among persons aged 50 and older is often related to factors such as living alone, the loss of family or friends, chronic illness, and sensory impairments [4]; however, individuals across the life span may experience social isolation and loneliness [7]. For example, Generation Z (age 18–22) has been reported as the loneliest generation [7]. COVID-19 also significantly increased young adults’ loneliness or decreased their social connectedness [8,9], which negatively influenced their mental health [10]. One study found that loneliness, rather than financial worries or concerns about the health impacts of COVID-19, was the single most important driver of reported emotional or mental health difficulties among young adults [11]. Moreover, the COVID-19 pandemic has also led to increased negative stereotypes about older adults [12].

One possible avenue for building and sustaining social connections in the community for older and younger generations is through befriending. Befriending is commonly defined as “a relationship between two or more individuals which is initiated, supported and monitored by an agency that has defined one or more parties as likely to benefit. Ideally the relationship is non-judgmental, mutual, purposeful, and there is a commitment over time” [13]. While there are diverse formats/platforms for befriending, intergenerational programs that connect younger and older adults are the most common. Intergenerational programs link older adults with younger generations across different age and educational levels, such as elementary students or college students. Intergenerational programs may include educational programs and art, information technology development, cultural heritage, health education, and therapeutic activities. As a nonpharmacological intervention, intergenerational programs have been utilized with promising results for both younger and older adults [14]. Systematic reviews with the most recent evidence-based programs/literature on intergenerational programs reported effectiveness in increased well-being and feeling of belonging among both younger and older participants [15,16], and showed intergenerational program benefits in improving quality of life and wellness, particularly among persons with Alzheimer’s disease and related dementias (ADRD) [14].

### 1.1. Big and Mini Program

To help older adults cope with social isolation or loneliness during COVID-19, a new intergenerational program, *Big and Mini*, was created in April 2020 to link young (ages 18+) and older adults (ages 50+) together (matched through a custom website developed for this program) in an effort to help increase social connectivity through weekly phone calls. The program designers wanted to be inclusive of age 50+ because the statistics from CDC [17] and the National Academies of Sciences, Engineering, and Medicine [4] showed that many adults aged 50 and older are socially isolated or lonely in ways that put their health at risk. The *Big and Mini* program (https://bigandmini.org/, accessed on 15 February 2022) was created and developed by three young adults. Since its launch, *Big and Mini* has successfully recruited over 6000 dyads of older and young adult participants. It also attracted attention from several media outlets [18].

Enrolling in *Big and Mini* proceeds as follows: Once a young or older adult is interested in participating, she/he/they can go to the *Big and Mini* website and fill out the required details on the signup page. Next, after submission, participants receive a verification email. By clicking the verification link in the email and logging onto *Big and Mini*, participants fill out their profile details so that the program can find them a match with similar interests, availability, and goals. Both *Big and Mini* participants receive online training details and complete a quiz at the end of the training, most of which are about the “do and do nots” during the program. Once a participant completes the training, she/he is qualified and approved to be matched. In the upcoming weeks, the program sends them an email with the details of their match. The participants then log onto the *Big and Mini* dashboard and schedule subsequent meetings based on an agreed date/time. When the time of their match arrives, they need to log in to their dashboard and click the meeting link to be redirected to the video call platform. 

The quality of interaction and sense of motivation between Bigs and Minis is of utmost importance to the program. One way to promote dyad activity is through the use of built-in conversation starters as well as guides explaining how the process works (one specialized for Bigs and one for Minis). Inherently, the serendipitous nature of matches (the idea that out of thousands of potential matches, they were created) also contributes to the motivation to continue making a meaningful effort to maintain the connection. Ultimately, if a decline in dyad activity continues, it may just be a sign that the match was not entirely compatible from the offset or that participants’ lives and schedules are changing. The program staff then look at the factors in the match, such as how the frequency of meetings changes over time and the individual’s backgrounds, to improve future matches. If a user requests another match, that will be accommodated accordingly.

### 1.2. The Present Study

A few months after the program launched, the *Big and Mini* program solicited feedback from participants to evaluate the usefulness of the program. Given that social isolation disproportionately affected older adults prior to the COVID-19 pandemic [4] as well as the negative effects of social distancing during COVID-19 [2], the *Big and Mini* developers wanted to specifically evaluate whether and how this new program helped with participants’ psychological well-being (e.g., loneliness, quality of life, and stress). In addition, there were growing concerns of ageism among young adults during COVID-19 and the team felt that it was important to know whether increasing social connectivity and connecting older and younger generations could combat ageism. 

The *Big and Mini* founders collaborated with a university research team for this study. Using a survey with close and open-ended questions, this team quantitatively evaluated dyad participants’ feedback on loneliness, satisfaction with life, as well as the young adult participants’ attitudes towards aging. The open-ended questions at the end of survey collected more detailed experiences from participants as well as participants’ feedback for the overall program. The overarching research questions for this study included:

#### 1.2.1. What Were the Experiences of Older and Young Adults in the *Big and Mini* Program? Specifically, the Study Aimed to Examine

What were the general psychological well-being conditions (loneliness, satisfaction with life, and stress levels compared to before COVID-19) and intergenerational closeness for Bigs and Minis, respectively?

Whether there were group differences of psychological well-being and intergenerational closeness between the Bigs and Minis?

What were the reasons and/or expectations of joining *Big and Mini*?

#### 1.2.2. What Were the Benefits and Lessons Learned While Participating in the Virtual Intergenerational Program, *Big and Mini*, for Dyads Participants? Especially, This Study Aimed to Explore

What did Bigs and Minis most like about or benefit from the program?

What did Bigs and Minis most dislike about the program?

## 2. Materials and Methods

### 2.1. Participants

At the time of the survey in September 2020, all current participants of the *Big and Mini* program were invited to complete the study survey. The study was approved by the Institutional Review Board (IRB) (#2021-0001) of University of Texas at Arlington. The informed consent form and survey questions were created through QuestionPro and the link was sent to participants’ email and advertised in the *Big and Mini* monthly news. The informed consent form was presented first in the QuestionPro survey. Participants were clearly informed of the purpose, study procedures, risks and benefits of participation, how deidentified data would be used for research, and that they could withdraw from participation at any stage of the study without any penalty. Contact information of the principal investigator and IRB was included in the consent form. Only after the participants signed the consent form by clicking the “accept” button could they move on to the survey. The survey consisted of several multiple-choice quantitative survey questions as well as three open questions at the end. To encourage a high response rate, participants were given the opportunity to enter a raffle for an Amazon e-gift card ($25 each, 2 winners for both *Big and Mini*). As per instructions from the IRB, interested participants for the raffle were asked to provide their email address in a separate QuestionPro survey. Among the active 120 dyads at the time of the study, 85 Bigs and 82 Minis started the survey. However, only 63 Bigs (age 50+) and 53 Minis (age 18+) completed the survey, yielding a 52.5% and 44.2% response rates for Bigs and Minis, respectively. 

### 2.2. Measures

#### 2.2.1. Quantitative Survey

**Loneliness**. This 6-item scale of the De Jong Gierveld [19] loneliness scale measured individual’s social and emotional loneliness. This instrument was validated across many countries and genders and ages, including older adults [19]. The items were responded based on a 5-point Likert scale from “1 = *none of the time*” to “5 = *all of the time*”. Item numbers 2, 3, and 5 were positive statements and thus the scores needed to be reversed. The sum of the six items ranged from 6 to 30, and the higher score meant a higher level of loneliness. The reliability of this scale was 0.81 for Bigs and 0.77 for Minis.

**Satisfaction with Life**. A 5-item satisfaction with life scale [20] was used to measure the Bigs’ cognitive judgements of their life satisfaction. This scale was shown to be a valid and reliable measure of life satisfaction, showing high internal consistency and reliability and being suited for use with different age groups and populations [21,22]. Each item was assessed on a 7-point Likert scale from “1 = *strongly disagree*” to “7 = *strongly agree*”. The sum of the five items contributed to the total score, ranging from 5 to 35. The higher the total score, the higher the life satisfaction. The value of Cronbach’s alpha was 0.93 for Bigs.

**Attitude towards Aging**. The Fraboni scale of ageism (FSA) [23] was used to assess the attitudes towards aging for the Minis only. The FSA has been extensively used among young adults and has shown high internal consistency and strong validity [24,25]. This scale has 29 items, each of which was responded by a 4-point Likert scale from “1 = *strongly disagree*” to “4 = *strongly agree*”. Items with positive statements were reversed. An additive score was calculated, ranging from 29 to 116. A higher score indicated a greater level of ageism. The Cronbach’s alpha was 0.85 for the Mini sample.

**Stress**. The stress compared to before COVID-19 was measured for both Bigs and Minis by a single question, “Compared to before the pandemic, how stressful is your life now?”, which was answered with either “1 = *higher than usual*”, “2 = *about the same*”, or “3 = *lower than usual*”. We dummy recoded the answers into “0 = *the same/lower than usual*” and “1 = *higher than usual*” for analyses.

**Other Key Variables**. Length in the *Big and Mini* program was a continuous variable assessing how many months the respondents had been in the program. Satisfaction with the program was measured based on a 5-point Likert scale from “1 = *very unsatisfied*” to “5 = *very satisfied*”. Intergenerational closeness was assessed by three questions adapted from the Intergenerational Solidarity Inventory [26]: namely, closeness with Big/Mini, good relationship with Big/Mini, and Big/Minis’ willingness to listen, each of which was measured from “1 = *not at all*”, “2 = *to some degree*”, to “3 = *very*”. An additive score was calculated for each respondent, ranging from 3 to 9, with a higher score indicating better intergenerational closeness. The reliability of this scale was 0.88 for Bigs and 0.90 for Minis.

#### 2.2.2. Qualitative Open Questions

At the end of the survey, both Bigs and Minis were asked three open-ended questions:

(1) Please explain why you chose to participate in the *Big and Mini* program? Or what are your expectations about this program?
(2) What aspect of the program that you like/benefit the best?
(3) What aspect of the program that you like the least?

### 2.3. Data Analyses

For the close-ended questions in the survey, quantitative data analyses were conducted. First, descriptives of the sample characteristics were conducted to have a brief understanding of the participants. Second, bivariate correlation analyses were performed, including all the key variables separately for Bigs and Minis. Subsequently, independent-sample t-tests were used to examine the differences of loneliness and intergenerational closeness between Bigs and Minis, and chi-squared test was conducted to compare the stress compared to before COVID-19 between Bigs and Minis. These analyses were performed using the Statistical Package for Social Sciences (SPSS) 25.0.

For the open-ended questions in the survey, content analysis was used to analyze the data by three members of the research team. The researchers, first, independently read the transcripts and developed codes and working definitions for each code [27]. Then, the team members met to group the codes into categories of related meaning and compared the codes and developed themes. The researchers discussed any disagreements until consensus was reached. As part of the analysis, the researchers avoided using preconceived categories and allowed the categories to come directly from the data [27]. As part of establishing trustworthiness, the data were triangulated with a third researcher in order to better understand the emergent themes and to avoid bias. Peer debriefing was also used to enhance credibility [28].

## 3. Results

### 3.1. Sample Characteristics

Table 1 shows the sample characteristics of the Bigs. The average age of the Bigs was 68.97 (*SD* = 7.30). Around half of them had a graduate or professional degree and the other half had some college or bachelor’s degrees. Most of the Bigs were women (73.0%) and White (87.3%). A total of 39.7% of the Bigs were married/cohabiting, followed by being divorced (25.4%), never married (15.9%), widowed (15.9%), and separated/others (3.2%). Nearly half of the Bigs lived alone, and another half lived with their spouse or others. The average score of the self-rated health for the Bigs was 3.65 out of 5 (*SD* = 0.96), reflecting a moderate level of health condition. The mean score of financial hardship was 1.40 out of 5, which indicated a low level of financial stress. Few of the Bigs had ADL (*M* = 0.02, *SD* = 0.13) or IADL (*M* = 0.65, *SD* = 1.28) assistance needs. The average length of the Bigs’s participation in the program was 3.44 months (*SD* = 2.09). Bigs reported a high level of satisfaction with the program (*M* = 3.65, *SD* = 1.44). Bigs had a moderate level of stress at the beginning of COVID-19 (*M* = 3.20, *SD* = 1.23). A total of 38.1% of the Bigs reported that their current stress level was higher than the stress before COVID-19. More than half of the Bigs did not receive any support to help them cope with stress during the pandemic, and 38.1% of the Bigs had received support a few times or once a week. The average score of social isolation for Bigs was 15.57 out of 30 (*SD* = 4.18), indicating a moderate level of social and emotional loneliness. The mean score of their life satisfaction was 23.79 out of 35 (*SD* = 7.92), reflecting a high level of satisfaction with life. Bigs reported close intergenerational relationships with Minis (*M* = 7.25, *SD* = 2.06).

The characteristics of the Minis are illustrated in Table 2. The average age of the Minis was 24.36 (*SD* = 7.79). A total of 84.9% of them had some college or higher-level degrees. Most of them were women (79.2%). A total of 45.3% of the Minis were White, followed by Asian (37.7%), and African American/Hispanic/others (15.1%). Most of them were never married (83.0%). A total of 20.8% of the Minis lived alone, and the remainder of the Minis lived with a roommate (30.2%), parent(s) (24.5%), or spouse/sibling/others (22.6%). Nearly half of them were full-time college students. The Minis reported a moderate level of health conditions (*M* = 3.79, *SD* = 0.96) and a low level of financial hardship (*M* = 1.60, *SD* = 0.85). Their average length in the program was 3.49 months (*SD* = 1.99). Minis reported a high level of satisfaction with the program (*M* = 3.96, *SD* = 1.17). They had a moderate level of stress at the beginning of COVID-19 (*M* = 2.48, *SD* = 1.00). A total of 37.7% of the Minis perceived higher stress than before COVID-19. A total of 34.0% of the Minis reported not receiving any support to cope with stress and 60.4% of the Minis reported receiving support a few times or once a week during the pandemic. The average score of social isolation for the Minis was 15.24 out of 30 (*SD* = 4.27), indicating a moderate level of social and emotional loneliness. Their mean score of ageism was 46.76 out of 116 (*SD* = 8.16), reflecting a good attitude towards aging. The Minis reported good intergenerational relationships with the Bigs (*M* = 7.00, *SD* = 2.08).

### 3.2. Quantitative Data Analyses

#### 3.2.1. Correlation among Key Variables

Table 3 summarizes the correlation analyses results among the key variables of the Bigs. Loneliness was negatively correlated with life satisfaction (*r* = −0.55, *p* < 0.001). Compared to experiencing ‘the same or lower stress than before COVID-19’, the perception of ‘higher stress compared to before COVID-19’ was correlated with a higher level of loneliness (*r* = 0.35, *p* < 0.01). Higher stress compared to before COVID-19 was related to lower life satisfaction (*r* = −0.37, *p* < 0.01). Intergenerational closeness was positively correlated with the Bigs’ satisfaction with the program (*r* = 0.58, *p* < 0.001).

The results of correlation analyses of the Minis are presented in Table 4. Length of participation in the program (*r* = −0.44, *p* < 0.01) and satisfaction with the program (*r* = −0.45, *p* < 0.01) were negatively correlated with the Minis’ ageism scores. Moreover, better intergenerational closeness was related to a lower level of ageism (*r* = −0.49, *p* < 0.01). Intergenerational closeness was positively correlated with the Minis’ satisfaction with the program (*r* = 0.84, *p* < 0.001).

#### 3.2.2. Group Comparisons on Key Measurements

In terms of the comparisons between Bigs and Minis, the average loneliness of the Bigs (*M* = 15.57, *SD* = 4.18) was slightly higher than that of the Minis (*M* = 15.24, *SD* = 4.26), but this difference did not reach statistical significance (*t* = 0.40, *p* = 0.687). A total of 38.1% of the Bigs reported higher stress compared to before COVID-19 and 38.5% of the Minis perceived a higher stress level than usual, which did not yield a significant result (*χ*^2^ = 0.00, *p* = 0.968). The average intergenerational closeness of the Bigs (*M* = 7.25, *SD* = 2.06) was higher than that of the Minis (*M* = 7.00, *SD* = 2.08), but such difference was statistically nonsignificant (*t* = 0.59, *p* = 0.557). Because no significant group differences were found for the key variables, a table was not created.

### 3.3. Qualitative Data Analyses

Themes from the content analysis suggested that the reasons to join or expectations of the program included: (1) mutually beneficial intergenerational connections; (2) making a difference during the Pandemic; (3) coping with loneliness; (4) friendship; and (5) curiosity. 

#### 3.3.1. Reasons/Expectations to Join the Program

**Mutually Beneficial Intergenerational Connections**. The most common theme related to joining *Big and Mini* was mutually beneficial intergenerational connections. For example, one Big mentioned, “I love, am interested in, connecting with young people” (B39) while another stated “it would be fun to get to know how a younger person feels about life… to have my perspective broadened” (B45). Similarly, another Big shared, “I wanted to connect with a young person, college student or young adult under 30, to discuss generation divide and how to improve that” (B28), while another Big reported, “I was curious and anxious to meet younger people to learn from them esp. their views on politics, learn how they are using technology, etc.” (B55). One Big shared:

“As an elder, I have always wanted to have a diverse life, including intergenerational connections. When I saw *Big and Mini*, I knew this not only could be a great way to stay connected to people in general because of COVID, but it could also fulfill my desire to be interacting with someone a lot younger than I and keep in the youth loop.”

(B4)

Similarly, Minis reported joining the program because of expectations of intergenerational connections. Two Minis said, “interested in intergenerational activities and learning (M9), “I wanted to get to know older members of the community, I don’t see my grandparents as often while in college and miss them” (M28). Another Mini shared:

“I typically enjoy older people and feel that intergenerational exchange is healthy for everyone involved. I wanted to be there for an older person who may need some company right now, and also experience the benefit of listening to and learning from them.”

(M45)

**Making A Difference During the Pandemic**. A second theme related to reasons for joining the *Big and Mini* program was to make a contribution to the program or other persons, especially during the COVID-19 pandemic. For Bigs, they wanted to share their life experience with Minis and hoped to mentor younger generations or support younger people. For example, two Bigs shared that “I had time and energy available and thought I might be able to help a young person”(B14) and “I like volunteering and was hoping to help someone” (B25). Others stated, “I love the idea of mentoring a young person” (B35), “Pass on hard learned experiences” (B40), and “I was looking to help a mini as far as business and life advice” (B42). Other Bigs similarly mentioned:

“I wanted to support young people who were experiencing social isolation and I also wanted to support the young people who took the initiative and created the program.”

(B17)

“I felt that I could share my experience in life—have lived and worked in 5 countries, have traveled to almost 50 countries, have been involved in charity projects and have been working for almost 50 years.”

(B12)

Minis also shared that they wanted to provide support to Bigs. Some Minis indicated that they wanted to positively impact others through this program, such as “I decided to sign up because I loved the idea of the one-on-one connection and because I knew that the platform was such a good opportunity to impact someone in a positive way” (M14). Some Minis specifically wanted to help older adults cope with loneliness by making connections with weekly calls. For example, one Mini said, “I wanted to help an elder feel like they have someone they can talk to and don’t have to feel lonely” (M20). 

While Minis wanted to make contributions to the society, the program, and specifically the older adults, they also expressed expectations of benefiting from connecting with the older generation, such as “To help me mature” (M31), “I like to be around people and learn” (M25), or “I thought it would be a great opportunity to learn from and interact with a person who has already experienced most of their life. I also was very interested in receiving advice from such a person” (M32). 

Some Minis hoped to make a difference in the Bigs’ lives while also benefiting from the intergenerational connections, as Minis said, “joining *Big and Mini* would be enjoyable for both me and my Big” (M24) or “I also needed support during the pandemic, and I usually help myself by helping others” (M36). Other Minis also mentioned:

“I joined because I thought it would be a great way to not only gain wisdom about what would be a good career path for me (as a fresh graduate with lots of decisions to make), but also because it’s incredibly important right now that the high-risk community does not feel lonely.”

(M13)

“I wanted to give back in some way during the pandemic, and this provided an easy option that was also beneficial for me. I’ve lost all of my grandparents, so having a connection with an older person was something that I was missing. I also strongly care about combatting loneliness in our society.”

(M16)

Another Mini shared:

“I’d like to do some volunteering over the summer, talk to someone who might experience loneliness because of their old age, tech unsavviness, or physical isolation. I’d like to learn something from a total stranger, too.”

(M33)

**Coping with Loneliness**. A third theme related to reasons to/expectations of joining the *Big and Mini* program was because of loneliness and wanting to cope with loneliness through this program. Bigs mentioned “boredom” (B18), “I am lonely” (B31), “Isolation and COVID now” (B47), “I have experienced loneliness and isolation. I have no family ties” (B34), and “My life came to a standstill once the pandemic arrived. I live alone, have no family in this state and no pets” (B16). Similarly, Mini participants shared, “I was looking for something to do at the start of the pandemic because everything just stopped. And I thought it would be nice to talk to someone since I was lonely.” (M4) and “I was feeling really lonely and sad and I wanted someone to talk to or to connect with” (M7). 

**Friendship**. A fourth theme was centered on friendship. Both Bigs and Minis mentioned expectations to meet new friends or to meet someone with a different life experience than them. For example, some Bigs shared “Make a new friend” (B41), “Wanted to meet someone ‘new’ to me & with different life experiences/opinions/perspective.” (B11), or “To meet someone new who I could connect with and keep me company”. One Big shared similarly, “I love meeting new people. New experience. New adventure. I always look for the adventures in life. No matter the age we all can learn from one another” (B13).

Minis also reported a desire for friendships and connection. For example, Minis said: “I wanted the chance to connect with someone who I wouldn’t have otherwise met. And to offer companionship during a hard time” (M3), “I wanted to meet someone new and keep people company” (M5), “To connect with others” (M17), “To meet others and connect with someone new during an isolating time” (M18), “Wanted to make a unique connection and meet someone I wouldn’t meet otherwise”, and “Make a new connection with someone who is older and has more life perspective than me” (M41). 

**Curiosity/Exploration**. A fifth theme was related to being curious about and wanting to explore the *Big and Mini* program. For example, some Bigs mentioned “out of curiosity” (B44), “To see how this nifty program works” (B2), and “I was curious about how it worked and wanted to share information with other older adults” (B56). Several Minis also shared that they simply wanted to explore the experience. For example, Minis stated “My mom thought it would be good for me” (M2), “Not exactly sure why, just kind of a spur of the moment choice” (M40), and “easy to register and connect. So just joined it” (M42).

#### 3.3.2. Best Liked Aspects of the *Big and Mini* Program 

Participants reported what aspects of the *Big and Mini* program that they liked best at the time of the survey. Themes included: intergenerational relationship and connections, learning and sharing, friendship, and the flexibility of the program. 

**Intergenerational Relationship and Connections**. Participants reported an appreciation for the intergenerational relationship and connections that the *Big and Mini* program offered. For example, one Big shared, “Just meeting and sharing with a delightful young lady the age of my grandchildren.” (B15) Another Big reported, “The conversations my Mini and I have. She reminds me of my granddaughter, and I really enjoy chatting with her.” (B59) 

Minis similarly shared:

“Connection of a young adult to an older adult and speaking and sharing your thoughts, knowledge, perspectives and mainly culture. And of course, meeting my BIG, was just the best thing ever.”

(M8)

“I love how open it is with my Big they did a wonderful job matching us. The chat function is also very nice so we can still talk during the week if we think of each other.”

(M4)

**Learning and Sharing**. Participants shared that they appreciated the learning and sharing opportunity that the *Big and Mini* program offered. First, both Bigs and Minis learned new things from their partners. For example, Minis shared, “I like learning about Jude’s (M3′s Big) life.” (M3) and “All the learning. You learn in a lot of ways and feel closer to someone that in any other way you wouldn’t have met” (M7). Similarly, learning new things from the younger generation was one of the benefits that Bigs perceived. As one Big shared below:

“Learning new things from my Mini, such as about the summers he spends in Peru with his cousins, Peruvians recipes (which I have cooked and enjoyed), information about ancient Roman history (his big interest), because he is bilingual, that has incentivized me to get serious about improving the limited Spanish I know (have a 150-day streak going on DuoLingo (have met my daily goal for 150 consecutive days), etc. My Mini has enriched my life in many ways. On our call this morning, we both lamented having to learn ‘Statistics’ in college.”

(B5)

Participants also mentioned that mutual learning from each other was an important component that they enjoyed about the *Big and Mini* program. For example, “I enjoy talking with my Big and we learn a lot from each other.” (M19), “Mentorship opportunities in both directions and simultaneously” (M13). Some Bigs even mentioned they liked the opportunity to provide help and give back to society. For example, one Big mentioned that she liked the “Opportunity to give back to an individual mini or a group—what I have learned from my experience in life in different continents.” (B12) Another Big had similar thoughts that she enjoyed offering help to her partner:

“My ‘mini’ is faithful in keeping our conversations going, and seems to be glad to talk with me. That means a lot. And I love getting to know her and being a listening, understanding—and maybe even helpful—ear.”

(B31)

**Friendship**. Friendship between two different generations was another component of the program that some participants liked best. Some Minis mentioned “I like that I have made a new friend and we continue to get closer each week.” (M24), “Meeting someone new who you wouldn’t have otherwise met” (M41), and “I feel like my and my Big have a lot in common and are a great match. He’s super easy to talk to!” (M16). Bigs had similar thoughts about friendship.

“I so enjoy my weekly meetings with my mini and that she e-mails me when she has some news. I actually feel that I am a part of her life now as she is a part of mine.”

(B7)

**Flexibility**. Some participants mentioned that they liked the flexibility and the virtual chat platform. For example, Minis talked about the flexible schedule: “Flexibility to log on to the meeting whenever is best for the Bigs and Minis. Also, the chat and nudge functions are useful.” (M26), “Ability to be flexible with my schedule” (M34), and “Being able to do good in a virtual format” (M47). Some participants also liked the *Big and Mini* program platform. For example, M17 and M43 as well as B44 and B60 commented they best liked the “Video chats” component of the *Big and Mini* program.

#### 3.3.3. Least Liked Aspects of the *Big and Mini* Program

When asking what aspects of the program that participants liked least, most of the participants did not provide a comment or shared positive comments instead. For example, several Bigs shared, “All is good.” (B3, B4) “So far everything has been beyond my expectations.” (B9)” I think it’s terrific, and I thank you for dreaming it up, implementing it, and continuing it” (B48). Some Bigs also gave positive feedback about the program: “…I think it’s wonderful and I’m so impressed that it was young people who conceived of it and created it. It was a brilliant idea. It was fun to be in two videos about it, too! “(B31) and “I’ve built a better sense of confidence about our youths by knowing her and seeing that she’s genuinely interested in engaging with me. Thank you.” (B32) Similarly, most Minis did not provide comments about what aspects of the program they disliked most. For example, Minis shared, “I don’t have anything to really complain about” (M4) or “Nothing” (B14, B18). However, two aspects were mentioned by some participants that they disliked most: technology and administration. 

**Technology (Internet connection)**. The aspects that participants disliked most was the technology, especially for Bigs. For example, Bigs shared: “Problems with the internet program. We now use zoom which seems to work better for us.” (B15), “Technology not always working when you need it.” (B17), “Technology not working. Poor connections on video chats.” (B57), “Technology at first was confusing” (B58), and “Video call sometimes glitches” (B59). Similarly, Minis reported technology issues such as “The video chat software doesn’t work too well but it’s okay my big and I FaceTime instead! ” (M23). Another Mini shared their experience:

“I think the online dashboard could be easier to use both for me and my Big. My Big wasn’t clear on exactly how to connect to our meeting, and we had trouble communicating on the dashboard because we never checked it. After my Big was a no-show the first time, I sent an email as instructed and never received a response. Thankfully my Big and I exchanged emails and were able to find a time to meet.”

(M16)

**Administration**. A few participants mentioned the administrative functions of the program that challenged them. For example, some participants thought that the matching process took longer than expected: “The long matching process. Still waiting to be matched with a new buddy because my old one didn’t show!” (M13). Some Bigs also disliked the matching process (e.g., Big 28). Another administration issue was the timing of each call, which was mentioned by the Bigs rather than the Minis. For example, one Big mentioned “Not knowing when to end the call” (B10), “no structure for ending the relationship” (B38). Lack of follow-up from staff or no communication with leaders was another challenge mentioned by some Bigs (e.g., B27, B51). 

## 4. Discussion

Using a survey with both closed and open-ended questions, this study evaluated dyad participants’ feedback of the *Big and Mini* program a few months after the program was launched. Both Bigs and Minis reported some level of social and emotional loneliness at the time of the survey when COVID-19 cases were rampant and there were no vaccines available yet to the public. However, they also reported high levels of satisfaction with life, satisfaction with the program, and intergenerational closeness. Content analysis of the open-ended questions showed that the reasons to join or expectations of the program were mainly friendship, mutually beneficial intergenerational connections, and coping with loneliness. Both Bigs and Minis also shared aspects of the program they liked best and least. In general, the results from both the closed and open-ended questions indicated that the *Big and Mini* program offers a promising approach with mutual benefits for both young and older generations. This confirmed previous research finding that intergenerational programs, as a nonpharmacological intervention, have been utilized with promising results for both young and older adults [14], especially for older participants [14,16].

First, consistent with previous research, the survey data showed that both the young and older generations experienced a certain level of stress and loneliness during COVID-19 when they joined the program and more than one-third of them even reported higher stress compared to before COVID-19. A national survey from Cigna reported that all ages may experience social isolation and loneliness [7]. The findings also corresponded to the prior literature showing high loneliness and/isolation and stress among older adults [3,29], as well as among young adults [8,9] during COVID-19. In addition, our study found that the Bigs experienced slightly higher levels of loneliness than the Minis. Though such differences were not statistically significant, this finding was not consistent with some of the literature that older adults reported lower stress and less negative emotions (e.g., loneliness) under the quarantine compared to younger adults [8,30,31]. The possible reasons might be that different measurements of loneliness and/or stress were used in the literature among different sample sizes.

Second, Bigs and Minis quantitatively reported high levels of satisfaction with life, satisfaction with the program, and intergenerational closeness. Specifically, intergenerational closeness with partners reported by both Bigs and Minis was highly corelated to their satisfaction with the program as well as lower levels of loneliness. These study findings echoed the prior literature that more intergenerational intimacy was associated with less COVID-19 pandemic-related stress and with more happiness and life satisfaction [32]. In addition, findings from this study also showed the benefits of lowering young adults’ ageism or negative image of older adults. Specifically, length of participation in the program, satisfaction with the program, and intergenerational closeness were all negatively correlated with Minis’ ageism scores. These findings are consistent with the previous literature that intergenerational programs between young and older generations may build on positive attitudes towards each other [33]. To cope with the constant repetition and consistency with ongoing negative age stereotyping before COVID-19 [34,35] as well as during COVID-19 [36,37], intergenerational program, such as *Big and Mini*, may provide a promising strategy to help young adults improve their views about aging. 

Third, the open-ended questions showed that the reasons to join/expectations of the program were friendship, mutually beneficial intergenerational connections, coping with loneliness, and curiosity. The most enjoyable parts of the program that participants reported corresponded to the expectations. These study findings are consistent with the previous systematic review that concluded that both the older adults and the youth gained a mutual benefit in the physical, social, and physiological domains through the intergenerational program [15]. Specifically, intergenerational programs are encouraging and promoting the idea of building intergenerational friendships. Scholars found that 68% of Americans have friends from different generations and that these intergenerational friendships can take on many forms of promoting meaningful friendships [38]. Empirical studies also found that intergenerational programs with older adults and children built on positive friendships for both participants [33], and intergenerational friendships promote the well-being of older adults and that these programs are important for the health of older adults and young people [39]. In addition, intergenerational programs also make connections for both young and older generations. A similar study linked older adults and college students and found that the participants enjoyed being a part of an intergenerational program since it promoted their well-being and social interactions as well as led them to have meaningful conversations with one another [40]. 

The COVID-19 pandemic has caused some grandchildren or grandparents to lose their physical, face-to-face connections. The *Big and Mini* program offers a platform for them as a way to cope with the loss of not seeing their grandkids or grandparents during COVID-19 and to expand their social network. Furthermore, the *Big and Mini* program has the potential to serve as a health promoter for participants to cope with loneliness. This is also consistent with the literature that showed that intergenerational programs decrease the risk of social isolation and give older adults a sense of meaning [41], especially during COVID-19 which has isolated most of the population. Since the pandemic, virtual intergeneration programs between isolated college students and older adults have helped participants to combat loneliness and isolation through connections [42]. Besides the benefits of friendship, mutually beneficial intergenerational connections, and coping with loneliness, both Bigs and Minis also mentioned a desire to contribute and help others. These findings corresponded to the literature that showed intergenerational programs make participants feel they have meaning and help others feel included, thus improving their self-esteem and quality of life [43]. 

### 4.1. Study Limitations

There were some limitations in this study. First, the survey was distributed to participants three months after the *Big and Mini* program was launched. Therefore, only cross-sectional data at one time point were collected and it is difficult to determine whether this intergenerational program is effective in coping with loneliness during COVID-19. Future studies should consider longitudinal surveys to track the psychological well-being of participants and investigate whether improvements on psychological wellbeing were achieved after participating in this program. Second, the survey questions, in general, were not in depth. For example, stress and satisfaction with life was asked by a single question, rather than a standardized scale. Third, though we tried to utilize a mixed methods approach by using both closed and open-ended questions, the answers to the open-ended questions were limited to their length and depth as well as a few without responses. Future studies should include conducting in-depth interviews with a random selection of participants in order to better understand their experiences with the *Big and Mini* program. Fourth, the small number of participants (achieved 64% of power based on post hoc power analyses), the relatively low response rate, and, therefore, the potential sample selection bias are also limitations of the present study. These limitations might influence the interpretation of the present study as people who take part in questionnaires are usually those who are more interested in the topic. Future studies need to examine why some Bigs and Minis started but did not complete the survey. Lastly, the sample of Bigs and Minis were generally healthy with high levels of education and with few concerns about financial needs. In addition, Bigs were those aged 50+, rather than 60+ or 65+ as suggested by the literature, though CDC suggests that those aged 50 are lonely. Therefore, the results from the present study as well as comparison with the previous literature should be interpreted with caution. Community outreach from the *Big and Mini* program should also include specific strategies for enrolling more diverse participants in terms of racial and ethnic background, socioeconomic status, and self-reported health.

### 4.2. Implications and Conclusions

Despite these limitations, the study suggested that the *Big and Mini* program is a promising approach for connecting young and older adults. In general, feedback from the Bigs and Minis showed that this program provides benefits of intergenerational learning and connection, friendship, coping with loneliness, and opportunities to contribute to and help others. These findings confirmed the overall benefits of intergenerational programs that link young and older generations together [44]. It would also be worthwhile to focus on the indicator of friendship between young and older generations in future research to deepen the knowledge in this area. As intergenerational programs have been on the rise, agencies that serve older adults and/or young adults may consider developing or creating new intergenerational programs, as well as promoting and extending existing programs. 

In addition, positive interaction and intergenerational relational qualities between young and older generations greatly matter. Some research suggests that simply increasing the frequency of contact between individuals is unlikely to reduce loneliness, unless these are positive, substantial connections, such as those among members of a “social support network” [11]. Therefore, moving forward, practitioners in different organizations should promote these types of intergenerational qualities in order to build deep and meaningful social connections, which in turn can increase positive psychosocial and health outcomes of both generations. 

Lastly, while virtual connections show promise in benefiting both young and older generations as shown in the *Big and Mini* program as well as in the literature [45], technology or internet connections have been a challenge for many older adults, especially during COVID-19 [42]. Bridging the digital divide is an ongoing concern that must be addressed through policy, research, and practice. 

## 5. Conclusions

The long-term social impact of social isolation and loneliness during COVID-19 remains largely unknown. As new variants emerge and vaccination rates stall or decline worldwide, we may continue to see increased rates of social isolation and loneliness across populations. It is imperative that interventions and programs seeking to promote meaningful social connectivity consider the continued need for virtual engagement due to safety and health concerns. The *Big and Mini* program offers a promising platform for promoting social connections, intergenerational closeness, and creating new friendships. Study findings bolster support for future research about the *Big and Mini* program and highlight the value of intergenerational relationships for other programs designed to combat social isolation and loneliness.

## Figures and Tables

**Table 1:** Sample characteristics of Bigs (*N* = 63).

Variables		*N*	Percentage	Mean (*SD*)	Range
Age		61		68.97 (7.30)	51–86
Educational level					
	Some college	14	22.2%		
	Bachelor’s degree	17	27.0%		
	Graduate or professional degree	32	50.8%		
Gender					
	Man	14	22.2%		
	Woman	46	73.0%		
	Other	1	1.6%		
Race					
	White	55	87.3%		
	Non-white	7	11.1%		
Marital status					
	Never married	10	15.9%		
	Married/cohabiting	25	39.7%		
	Widowed	10	15.9%		
	Divorced	16	25.4%		
	Separated/Others	2	3.2%		
People who live together now					
	Live alone	31	49.2%		
	Live with spouse	19	30.2%		
	Live with others	10	15.9%		
Self-rated health		62		3.65 (0.96)	1–5
Financial hardship		62		1.40 (0.66)	1–5
ADL		60		0.02 (0.13)	0–7
IADL		62		0.65 (1.28)	0–8
Length in the program		59		3.44 (2.09)	0–8
Satisfaction with the program		60		3.65 (1.44)	1–5
Stress at the beginning of COVID-19		60		3.20 (1.23)	1–5
Stress compared to before COVID-19					
	Higher than usual	24	38.1%		
	About the same	30	47.6%		
	Lower than usual	9	14.3%		
Frequency of support					
	Never	35	55.6%		
	A few times	17	27.0%		
	Once a week or less	7	11.1%		
	Several times a week	4	6.3%		
Loneliness		60		15.57 (4.18)	6–30
Satisfaction with life		57		23.79 (7.92)	5–35
Intergenerational closeness		53		7.25 (2.06)	3–9

*SD*, standard deviation. ADL, activities of daily living. IADL, instrumental activities of daily living.

**Table 2:** Sample Characteristics of Minis (*N* = 53).

Variables		*N*	Percentage	Mean (*SD*)	Range
Age		45		24.36 (7.79)	18–50
Educational level					
	Less than High School	4	7.5%		
	High School	3	5.7%		
	Some College	29	54.7%		
	Bachelor’s Degree	7	13.2%		
	Graduate or Professional Degree	9	17.0%		
Gender					
	Man	10	18.9%		
	Woman	42	79.2%		
Race					
	White	24	45.3%		
	Asian	20	37.7%		
	African American/Hispanic/Others	8	15.1%		
Marital status					
	Never married	44	83.0%		
	Married/cohabiting	6	11.3%		
	Others	2	3.8%		
People who live together now					
	Live alone	11	20.8%		
	Live with parent(s)	13	24.5%		
	Live with roommate	16	30.2%		
	Live with spouse/Sibling/Others	12	22.6%		
Employment					
	Full-time college student	25	47.2%		
	Full-time employee	10	18.9%		
	Part-time employee/part-time student	5	9.4%		
	Others	12	22.6%		
Self-rated health		52		3.79 (0.96)	1–5
Financial hardship		52		1.60 (0.85)	1–5
Length in program		49		3.49 (1.99)	0–8
Satisfaction with program		51		3.96 (1.17)	1–5
Stress at the beginning of COVID-19		52		2.48 (1.00)	1–5
Stress compared to before COVID-19					
	Higher than usual	20	37.7%		
	About the same	28	52.8%		
	Lower than usual	4	7.5%		
Frequency of support					
	Never	18	34.0%		
	A few times since pandemic	21	39.6%		
	Once a week or less	11	20.8%		
	Several times a week	2	3.8%		
Loneliness		50		15.24 (4.27)	6–30
Attitude towards aging		46		46.76 (8.16)	29–116
Intergenerational closeness		46		7.00 (2.08)	3–9

*SD*, standard deviation.

**Table 3:** Correlations among key variables for Bigs.

	1	2	3	4	5	6
1. Loneliness	1					
2. Life satisfaction	−0.55 ***	1				
3. High stress compared to before COVID-19	0.35 **	−0.37 **	1			
4. Length in program	−0.00	0.05	0.02	1		
5. Program satisfaction	0.22	0.18	0.00	0.25	1	
6. Intergenerational closeness	−0.20	0.18	−0.12	0.24	0.58 ***	1

Note. ** *p* < 0.01, *** *p* < 0.001.

**Table 4:** Correlations among key variables for Minis.

	1	2	3	4	5	6
1. Loneliness	1					
2. Ageism	0.19	1				
3. High stress compared to before COVID-19	0.12	−0.08	1			
4. Length in program	−0.08	−0.44 **	0.29 *	1		
5. Program satisfaction	−0.16	−0.45 **	0.10	0.07	1	
6. Intergenerational closeness	−0.11	−0.49 **	0.00	0.22	0.84 ***	1

Note. * *p* < 0.05, ** *p* < 0.01, *** *p* < 0.001.

## Data Availability

The date can be found at https://mavsuta-my.sharepoint.com/:f:/r/pesonal/lingxu_uta_edu/Docments/Big%20and%20Mini%20data?csf=1&web=1&e=jGNj3n (accessed on 15 February 2022).
